# Comprehensive Investigation of Constant Voltage Stress Time-Dependent Breakdown and Cycle-to-Breakdown Reliability in Y-Doped and Si-Doped HfO_2_ Metal-Ferroelectric-Metal Memory

**DOI:** 10.3390/nano13142104

**Published:** 2023-07-19

**Authors:** Ting-Yu Chang, Kuan-Chi Wang, Hsien-Yang Liu, Jing-Hua Hseun, Wei-Cheng Peng, Nicolò Ronchi, Umberto Celano, Kaustuv Banerjee, Jan Van Houdt, Tian-Li Wu

**Affiliations:** 1 International College of Semiconductor Technology, National Yang Ming Chiao Tung University, Hsinchu 30010, Taiwan; emmatony11111.st10@nycu.edu.tw (T.-Y.C.); kcwang.st09@nycu.edu.tw (K.-C.W.); wcpeng.st11@nycu.edu.tw (W.-C.P.); 2 Institute of Electronics, National Yang Ming Chiao Tung University, Hsinchu 30010, Taiwan; hyliu.ee10@nycu.edu.tw; 3 Institute of Pioneer Semiconductor Innovation, National Yang Ming Chiao Tung University, Hsinchu 30010, Taiwan; jhhsuen.10@nycu.edu.tw; 4 Imec, 3000 Leuven, Belgium; nicolo.ronchi@imec.be (N.R.); umberto.celano@asu.edu (U.C.); kaustuv.banerjee@imec.be (K.B.); jan.vanhoudt@imec.be (J.V.H.); 5 School of Electrical, Computer and Energy Engineering, Arizona State University, Tempe, AZ 85287, USA; 6 Department of Physics and Astronomy, KU Leuven, 3000 Leuven, Belgium

**Keywords:** ferroelectric, domain size, reliability

## Abstract

In this study, we comprehensively investigate the constant voltage stress (CVS) time-dependent breakdown and cycle-to-breakdown while considering metal-ferroelectric-metal (MFM) memory, which has distinct domain sizes induced by different doping species, i.e., Yttrium (Y) (Sample A) and Silicon (Si) (Sample B). Firstly, Y-doped and Si-doped HfO_2_ MFM devices exhibit domain sizes of 5.64 nm and 12.47 nm, respectively. Secondly, Si-doped HfO_2_ MFM devices (Sample B) have better CVS time-dependent breakdown and cycle-to-breakdown stability than Y-doped HfO_2_ MFM devices (Sample A). Therefore, a larger domain size showing higher extrapolated voltage under CVS time-dependent breakdown and cycle-to-breakdown evaluations was observed, indicating that the domain size crucially impacts the stability of MFM memory.

## 1. Introduction

Since the initial discovery of Si-doped HfO_2_ materials’ ferroelectric properties in 2011 [1], significant attention has been directed toward oxide materials with a fluorite structure, such as doped HfO_2_ [2], and the solid solution of Hf_x_Zr_1-x_O (HZO) [3]. These materials have garnered interest for their compatibility with advanced process technology and ability to exhibit ferroelectric behavior even at thicknesses of ≤10 nm, setting them apart from traditional perovskite ferroelectric materials. Recent studies have revealed that the thickness of HfO_2_-based ferroelectric films can be reduced to as little as 1 nm while maintaining the occurrence of spontaneous polarization and its ability to alter polarization direction [4]. This finding suggests that HfO_2_-based ferroelectric film does not have a critical threshold for scaling down, unlike perovskite materials. This exceptional scalability feature indicates a promising advantage for developing memory devices driven by polarization.

Furthermore, ferroelectric HfO_2_-based technologies are promising materials for nonvolatile memories [5], logic FETs [6], and neuromorphic applications [7,8,9] because of their compatibility with complementary metal-oxide-semiconductor (CMOS) technology [10,11]. Ferroelectric properties can be induced by various doping species in HfO_2_ films, e.g., Zr, Si, Al, Gd, etc. Recently, high-performance ferroelectric-based technologies have been demonstrated with optimized annealing conditions, dopants, electrodes, interfacial layers, etc. [12,13,14,15,16,17,18,19,20,21]. However, reliability remains one of the main concerns in ferroelectric-based technologies [22], particularly the instability related to domain size’s impact on time-dependent dielectric breakdown and cycle-to-breakdown. Understanding the impact of domain size on the stability of ferroelectric-based devices is not extensively reported in the literature.

In this study, Yttrium (Y)-doped and Silicon (Si)-doped HfO_2_ metal-ferroelectric-metal (MFM) devices were fabricated to intentionally induce different domain sizes in metal-ferroelectric-metal (MFM) devices. The o-phase with ferroelectricity can be induced through the annealing process of differently doped ferroelectric films since the crystal radius of doping below/above Hf can stabilize the t-/c-phases [2]. CVS time-dependent stress and cycle-to-breakdown measurements were conducted. Furthermore, the correlations between domain size and CVS time-dependent stress and cycle-to-breakdown stability are discussed and analyzed to understand the impact of domain size.

## 2. Materials and Methods

**Figure 1:**
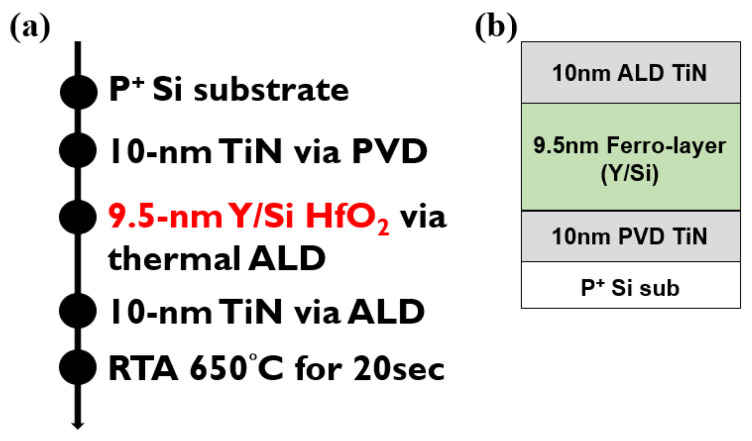
(**a**) Schematic of the process flow and (**b**) schematic structure of MFM capacitors with different dopants in ferroelectric layers.

Figure 1 shows the schematic structure of metal-ferroelectric-metal (MFM) capacitors and a brief process flow of this work. At first, 10-nm TiN was deposited via PVD as the bottom electrode. Next, 9.5-nm HfO_2_-based ferroelectric layers with two different dopants, Y and Si, were deposited via thermal ALD at 300 °C. Afterward, another 10-nm TiN was deposited via ALD on the ferroelectric films as the top metal electrode. Lastly, RTA was conducted in N_2_ ambient at 650 °C for crystallization for 20 s.

The measurement setup for the capacitor used in this study was 2400 μm^2^ (60 μm × 80 μm). To perform electrical characterizations such as I-V (current-voltage) and time-dependent dielectric breakdown (TDDB) measurements, a Keysight B1500 Source Measurement Unit (SMU) Keysight, USA, was employed. To characterize the ferroelectric properties, including P-V (polarization-voltage) and cycle-to-breakdown measurements, a Keysight B1530 Waveform Generator/Function Measurement Unit (WGFMU) Keysight, USA, was utilized. In this setup, the capacitor was biased at the bottom via a chuck electrode, while a ground electrode was placed on top.

**Figure 2:**
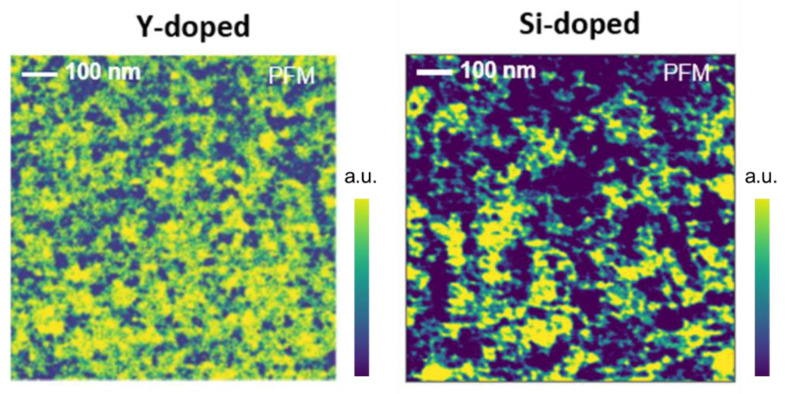
Contact resonance piezoresponse force microscopy PFM measurements in Sample A (Y-doped) and Sample B (Si-doped).

To compare the sample’s domain size, distribution, and homogeneity, we used contact resonance piezoresponse force microscopy (PFM). Although a quantitative interpretation of the results is beyond the scope of this work, we used the same probe. We also operated under the same conditions on two samples with the same physical thickness (9.5 nm). Therefore, the results represent a relative comparison between samples and can be used to analyze the domain size (Figure 2). For instance, Sample A (Y-doped) has a smaller domain size than Sample B (Si-doped). Details of the domain structures can also be found elsewhere [23]. Table 1 briefly describes the dopants and domain sizes used in this study. Distinct differences in the domain sizes of Sample A and Sample B can be used to understand the impact of domain size on the reliability of MFM devices.

## 3. Results

**Figure 3:**
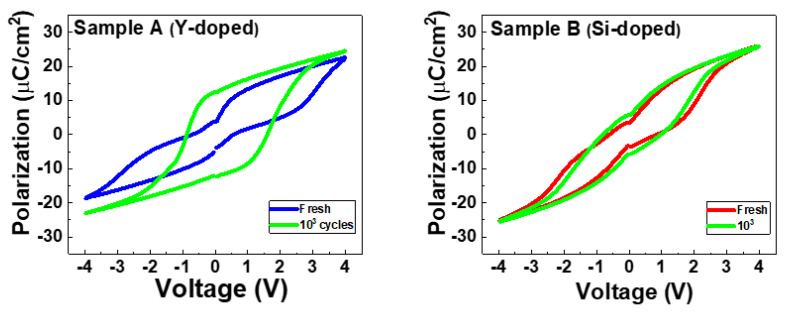
P-V characteristics in Samples A and B.

**Figure 4:**
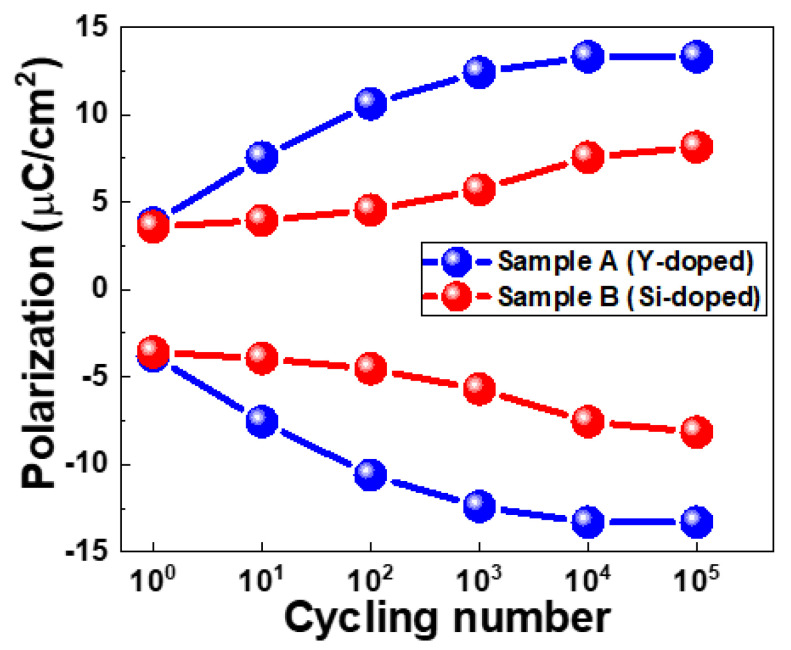
Polarization characteristics with respect to cycling numbers.

To understand ferroelectricity, we used P-V measurements with a triangular pulse of 10 μs/V and a trapezoidal plus with T_r_ (rising time)/T_f_ (falling time) fixed at 0.5 μs and T_width_ (pulse width) set at 1μs for the cycling. Figure 3 shows the P-V characteristics of Samples A and B in the fresh state and after 10^3^ cycles. Figure 4 shows comprehensive endurance characteristics at different cycling numbers. Sample A shows a slightly larger 2Pr than Sample B in the pristine state. Upon increasing the cycling number, Sample A exhibits a clear wake-up effect with a saturation of 2Pr after 10^4^ cycles. However, Sample B does not exhibit a saturation of 2Pr. Overall, Sample A shows a larger 2Pr than Sample B after 10^5^ cycles

**Figure 5:**
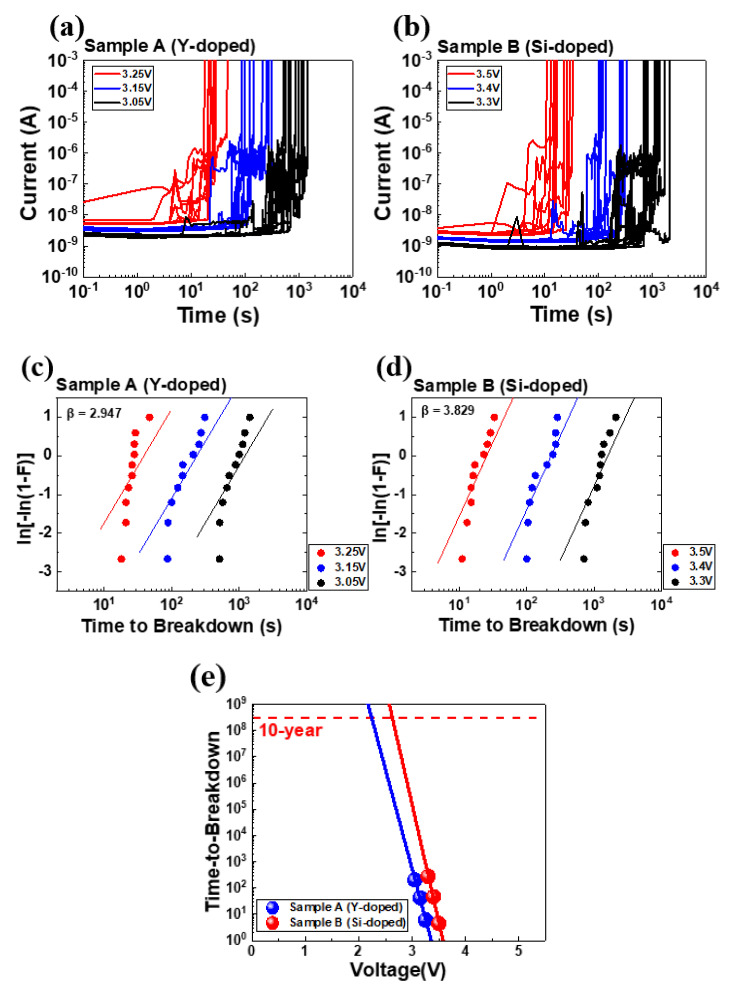
Gate current for (**a**) Sample A and (**b**) Sample B, as monitored by six different voltages (10 devices per group). (**c**,**d**) show corresponding Weibull plots of t_BD_. (**e**) Operating voltage extrapolation for a 10-year lifetime at 1% failure for devices with Samples A and B.

**Figure 6:**
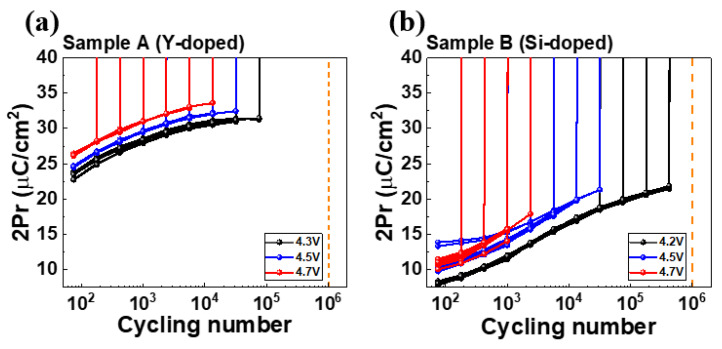

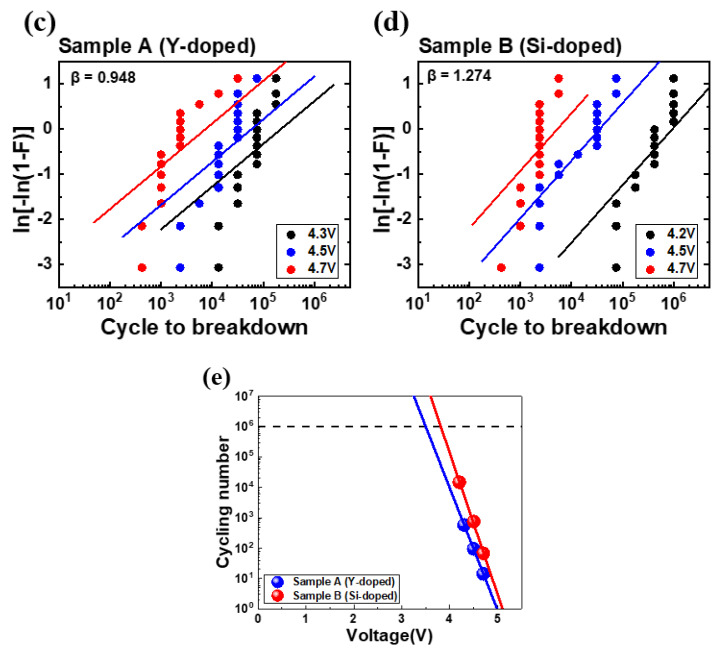
2Pr for (**a**) Sample A and (**b**) Sample B, as monitored by six different voltages (10 devices per group). (**c**,**d**) show corresponding Weibull plots of cycle-to-breakdown. (**e**) Operating cycling number extrapolation for a 10^6^-cycling lifetime at 1% failure for Samples A and B.

To understand the impact of domain size on time-dependent breakdown stability, we performed constant voltage stress time-dependent dielectric breakdown and cycling-to-breakdown evaluations. Figure 5 and Figure 6 show the results of the constant voltage stress (CVS) time-dependent dielectric breakdown (TDDB) and cycle-to-breakdown evaluations in Samples A and B, respectively.

Figure 5c,d shows Weibull plots of time-to-breakdown (t_BD_) distributions for three TDDB VG conditions, which follow the Weibull failure distribution:

(1) $$\ln\left[-\ln\left(1-F\left(t\right)\right)\right]=\beta \ln\left(t\right)-\beta \ln\left(\eta \right)$$

where *t* is the time; *β* is the shape parameter; *η* is the scale factor of 63.2% value. The fitted *β* is 2.497 and 3.829 for Samples A and B, respectively. A higher *β* implies a tight distribution and small variability.

In Figure 5e, lifetime of 1% failure analyses are extrapolated from the Weibull plot of t_BD_ distribution and projected to a 10-year line. The operating voltages of Sample A are slightly lower than Sample B (2.62 V) at 2.24 V.

In designing the cycle-to-breakdown (Cycle-to-BD) measurement, we chose a PUND waveform with triangular pulses for the reading state. We set the rising, falling, and delay times (T_r_/T_f_/T_delay_) to a fixed duration of 5 μs. The PUND waveform allowed for clear observation of whether or not the sample experienced breakdown. In the cycling state, trapezoidal pulses were used as the waveform. The T_r_ and T_f_ were fixed at 0.5 μs, while the T_width_ was set to 1 μs. To determine the cycles of the chosen reading step, we divided the interval to reach 1E6 cycles into 12 segments. After calculations, we determined that 10^0.375^ cycles would serve as the interval between the two consecutive reading states.

Similar to the TDDB analysis, cycle-to-breakdown (Cycle-to-BD) distributions for three different Cycle-to-BD VG conditions were used to construct Weibull plots. These plots were then used to extract fitting values from the ß value and generate lifetime curves. However, to adapt the Weibull failure distribution to the Cycle-to-BD analysis, the time-to-BD (t_BD_) was transformed into cycle-to-BD (C_BD_). Figure 6c,d display the fitting β values and corresponding lines for different devices. Sample A yielded a fitted β value of 0.948, while sample B had a βvalue of 1.274. These values are consistent with the TDDB analysis results, where Sample B exhibited a higher β value. The discrepancy in β values between TDDB and Cycle-to-BD measurements may be due to the Cycle-to-BD measurement using both positive and negative pulses during the cycling stage compared to TDDB measurements with a constant positive bias.

Figure 6e illustrates the 1% failure analysis of the 10^6^-cycle lifetime analysis. The trend observed in the Cycle-to-BD analysis is similar to the TDDB analysis. For Sample A, the determined operating voltage is 3.45 V, which is slightly lower than Sample B’s operating voltage of 3.79 V.

**Figure 7:**
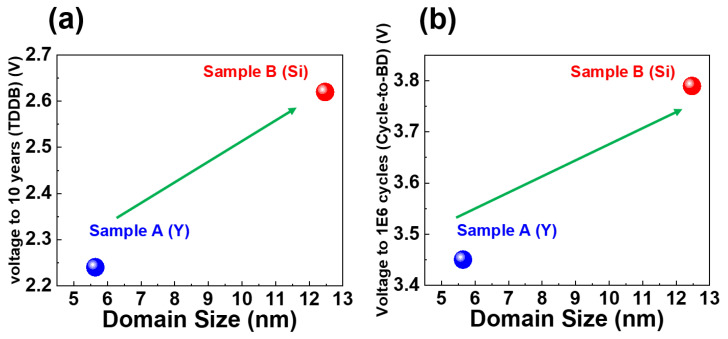
Extrapolated voltages vs. domain size under CVS TDDB (**a**) and cycle-to-breakdown measurements (**b**).

Figure 7 shows the correlation between operation voltage (based on constant voltage stress TDDB and cycle-to-breakdown measurements) and domain size. Table 2 summarizes operation voltages based on CVS TDDB and cycle-to-breakdown evaluations. Figure 7 indicates that a larger domain size exhibits better TDDB and cycle-to-breakdown stability, i.e., higher 10-year operation voltages and higher voltages up to 10^6^ cycles. In addition, Table 3 presents the maximum lifetime of targeting applied voltage at 3V results that Samples A and B can withstand in seconds. We calculated the lifetime of the Cycle-to-BD measurement by multiplying the predicted breakdown cycling number by a duration of 1 cycle in the measurement waveform design. According to our results, the lifetime measured in the TDDB analysis is longer than in the Cycle-to-BD analysis. This finding indicates that the measurement technique involving the continuous application of the same bias direction is less likely to induce defects generation and device breakdown than the technique involving a continuously changing bias direction. However, from the lifetimes in seconds, Sample B still exhibits a longer lifetime with a larger domain size in both the TDDB and Cycle-to-BD measurements. These findings are consistent with previous results.

**Figure 8:**
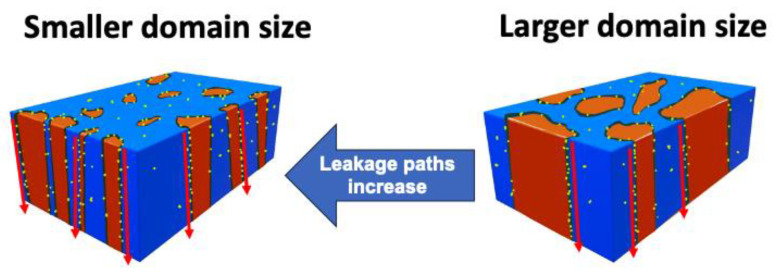
Schematic illustration of domain size’s effect on the leakage path.

The charging effects along the domain boundaries have been reported [24]. Therefore, larger domain sizes represent fewer domain boundaries (Figure 8) and reduce the chance of charging effects that create leakage paths as bias is applied to the device, thereby improving TDDB and cycle-to-breakdown stability. Besides, it is worth noting that Sample B exhibits larger β than Sample A, indicating better uniformity due to fewer domain boundaries in Sample B. In summary, larger domain sizes and fewer domain boundaries can improve CVS TDDB and cycle-to-breakdown reliability. However, samples with a larger domain size may degrade ferroelectricity.

## 4. Conclusions

In this study, the impact of domain size on the constant voltage stress TDDB and cycle-to-breakdown reliability are systematically reported. Firstly, MFM devices were fabricated with two different dopants in the ferroelectric layer, which intentionally induced different domain sizes. The PFM analysis indicated that Sample B (Si-doped) had a larger domain size than Sample A (Y-doped). Furthermore, CVS TDDB and cycle-to-breakdown evaluations were conducted in Sample A and Sample B, indicating that Sample B had better CVS TDDB and cycle-to-breakdown stability. A clear correlation was observed between the larger domain size and better time-dependent stability, which may be attributed to fewer domain boundaries in Sample B. We are the first to report the effects of domain size on CVS TDDB and cycle-to-breakdown reliability and conclude that optimizing the domain size can improve devices’ reliability.

## Figures and Tables

**Table 1:** A brief summary of the dopants and domain sizes used in this study.

	Dopant in Ferroelectric Layer	Domain Size (nm)	Doping Concentration
**Sample A**	Y	5.6	2.9%
**Sample B**	Si	12.5	2.5%

**Table 2:** Summary of the extrapolated operation voltage.

	10-year Operation Voltage Based on CVS TDDB	10^6^-Cycling Operation Voltage Based on Cycle-to-Breakdown
**Sample A**	2.24	3.45
**Sample B**	2.62	3.79

**Table 3:** The maximum lifetime results that two samples can withstand in seconds.

	Lifetime of Cycle-to-BD @3V (s)	Lifetime of TDDB @3V (s)
**Sample A**	203	515
**Sample B**	12,170	146,058

## Data Availability

The data presented in this study are available upon request from the corresponding author. The data are not publicly available due to privacy.
